# 2% Ganciclovir Eye Drops Control Posner-Schlossman Syndrome Relapses With/Without Cytomegalovirus Intraocular Reactivation

**DOI:** 10.3389/fmed.2022.848820

**Published:** 2022-03-09

**Authors:** Qilian Sheng, Ruyi Zhai, Xintong Fan, Xiangmei Kong

**Affiliations:** ^1^Eye Institute and Department of Ophthalmology, Eye & ENT Hospital, Fudan University, Shanghai, China; ^2^National Health Commission Key Laboratory of Myopia (Fudan University), Key Laboratory of Myopia, Chinese Academy of Medical Sciences, Shanghai, China; ^3^Shanghai Key Laboratory of Visual Impairment and Restoration, Shanghai, China

**Keywords:** topical ganciclovir treatment, cytomegalovirus infections, Posner-Schlossman Syndrome, ophthalmic solutions, antiviral therapy

## Abstract

**Background:**

To observe and compare the efficacy of 2% ganciclovir eye drops in the treatment of Posner-Schlossman Syndrome relapses with/without cytomegalovirus intraocular reactivation.

**Methods:**

A prospective cohort study enrolling 101 patients diagnosed unilateral Posner-Schlossman Syndrome in Eye & ENT hospital, Fudan University, Shanghai, China. Thorough ophthalmic examinations were given. Aqueous humor sample was collected from the attacked eye of each patient and all pathogen immunoglobulins tests were performed. All patients were treated with appropriate corticosteroids and intraocular pressure-lowering drugs. 2% ganciclovir eye drops were given to patients whose cytomegalovirus antibody aqueous humor/serum correction ratio >0. Patients were followed up for 2 months. Ocular manifestations and cumulative drug dose were recorded.

**Results:**

A cytomegalovirus ratio >0.40 was considered cytomegalovirus reactivation. The reactivation group (*N* = 46) had significantly higher percent of iris depigmentation (78.26%, *P* < 0.05) and endothelial cell loss rate (19.46%, *P* < 0.001) than the latent group (*N* = 55, 58.18% and 10.86%, respectively). The cumulative treatment time and 2% ganciclovir doses were 6.50 ± 4.67 weeks and 181.70 ± 130.95 drops for the reactivation group; 5.95 ± 4.11 weeks and 161.89 ± 110.66 drops for the latent group (*P* > 0.05). The median cumulative 2% ganciclovir estimated for inflammation control were 252.00 ± 50.71 and 224.00 ± 32.45 drops for the reactivation and latent group. The residual rate of uncontrolled cases was 0.19 ± 0.15 and 0.00, respectively (*P* < 0.05).

**Conclusions:**

A treatment course of 8–9 weeks' 2% ganciclovir is recommended to relapses both with and without cytomegalovirus intraocular reactivation. Preventive ganciclovir application may benefit patients with historical cytomegalovirus infections.

**Clinical Trial Registration:**

www.chictr.org.cn, identifier: ChiCTR1900022340, Date: 2019/04/06.

## Introduction

Posner-Schlossman Syndrome (PSS), also known as glaucomatocyclitic crisis, is characterized by recurrent unilateral attacks of anterior non-granulomatous uveitis and elevated intraocular pressure (IOP) (1, 2). Large granulomatous keratic precipitates (KPs) and loss of corneal endothelial cells are commonly seen (3). Though the etiology of PSS has not been fully elucidated, a bulk of literature supports the cytomegalovirus (CMV) as the leading cause of anterior chamber infection (4–6). In immunocompetent eyes, CMV replication is restricted in the anterior segment, presented as chronic anterior uveitis or recurrent episodic iritis with raised IOP, much resembling PSS (7, 8). The replication of latent CMV poses a continuous threat to anterior segment (9, 10). To further investigate the manifestations and pathogenesis, a comparative observation of PSS relapses with/without CMV reactivation is conducted in our center.

Evidence is now emerging that inhibiting CMV replication from the anterior chamber with antiviral therapy leads to improved disease control. Various antiviral agents have been tested, such as oral valganciclovir, 0.15% ganciclovir gel, ganciclovir intravitreal injection, etc. (11–14). It appears that 2% ganciclovir concentration helped maintaining good prognosis (15, 16). We are aiming at proposing detailed treatment schedule for 2% ganciclovir during acute relapses in this study.

## Materials and Methods

### Participants

This study was approved by the Ethics Committee of Eye & ENT Hospital, Fudan University and was consistent with Helsinki Declaration. From 2019 to 2020, 101 patients diagnosed PSS in Eye & ENT Hospital, Fudan University, Shanghai, China were enrolled in our study. Informed consent was obtained from each participant. The inclusion criteria were as follows: (1) recurrent attacks of mild, unilateral, non-granulomatous anterior uveitis accompanied by markedly elevated IOP, small white KPs on the endothelial surface of the central cornea, open angle, no posterior synechia, and no inflammatory lesions in the posterior segment of the eye; (2) the IOP and anterior chamber inflammation returned normal between attacks; (3) patients had to be in the attack phase of PSS, and a complete ocular examination of this condition was performed as the baseline information; (4) CMV immunoglobulin G (IgG) aqueous humor/serum albumin concentration correction ratio >0.

The exclusion criteria were as follows: (1) primary glaucoma or elevated IOP caused by other known factors; (2) previous trauma, uveitis caused by herpes simplex virus or herpes zoster virus, retinal or corneal diseases; (3) allergic to any corticosteroids, IOP-lowering drugs or ganciclovir; (4) liver or kidney dysfunction; (5) pregnant or lactating women.

All patients underwent thorough ophthalmic examinations and detailed medication histories were recorded. Anterior chamber of the affected eye was tapped when the patient presented with a new episode of hypertension uveitis, prior to starting new medication. All the patients underwent anterior chamber paracentesis only once. After obtaining the informed consent, anterior chamber tap was performed using an aseptic technique under slit lamp (Zeiss, Germany). Paired serum and aqueous humor samples were assayed to detect CMV IgG by enzyme-linked immunosorbent assay (ELISA, Virion/Serion, Germany) as described by Wang XL et al. (17). The total amount of IgG in aqueous humor and serum was determined by a radial immunodiffusion technique. The aqueous humor/serum albumin concentration correction ratio was calculated as previously described (17). Other possible pathogen antibodies were also tested for, including herpes virus 1 and 2, varicella zoster virus, rubella virus and toxoplasma gondii.

All patients were treated with corticosteroids [mainly 1% prednisolone (Allergan Pharmaceuticals, Ireland), 0.1% dexamethasone (ALCON-COUVREUR, Belgium), or 0.1% fluorometholone (Santen Pharmaceuticals, Japan)] and IOP-lowering drugs (mainly beta-blockers, alpha-2 adrenergic agonists or carbonic anhydrase inhibitors). If IOP of the attacked eye lowered below 21 mmHg and anterior chamber inflammation reduced obviously compared to last visit (e.g., KPs, anterior chamber cells, corneal edema etc.), steroids were tapered off to two times daily, once daily, once every other day every week and were finally stopped. 2% ganciclovir eyedrops were used four times daily. The cumulative drug dose was calculated by daily dosage and duration.

We set a mean follow-up period of 6 weeks, which showed a short-term efficacy of 2% ganciclovir eye drops in our previous study (18). In case some patients lived far away or may have special occasions, we recorded the actual follow-up periods and presented them as mean ± standard deviation (SD).

The number of fresh KPs in the anterior chamber was graded in four degrees: 0: no fresh KP in the anterior chamber; I: 1–5 KPs; II: 6–10 KPs; III: >10 KPs. The old KPs were classified in 0 (old KP absent) and 1 (old KP present). Iris depigmentation was confirmed when obvious color fade and various degrees of moth-eaten appearance were observed under slit lamp.

### Preparation of 2% Ganciclovir Eye Drops

0.25 g dry powder of ganciclovir for injection (Luoxin, Shandong Province, China) was dissolved in 2.5 ml water for injection (GIBCO WFI, Thermo Fisher Scientific, USA); 1.25 ml ganciclovir solution was mixed with 5 ml 0.3% Sodium Hyaluronate Eye Drops (Santen, Japan). By this method, 125 mg:6.25 ml (2%) ganciclovir eye drops are made. The 2% ganciclovir eye drops were prepared by Pharmacy of Eye & ENT Hospital (qualified to prepare clinical solutions) and approved by Ethics Committee of Eye & ENT Hospital, Fudan University.

### Statistical Analysis

Statistical analysis was performed with SPSS version 21.0 software. Student *t*-tests were performed to compare IOP level and drugs dosage between groups; χ^2^ tests were performed to compare KP and inflammation manifestations. Kaplan-Meier survival analysis was used to compare treatment outcome between CMV reactivation and latent group. A *P*-value less than 0.05 was considered significant.

## Results

**Figure 1:**
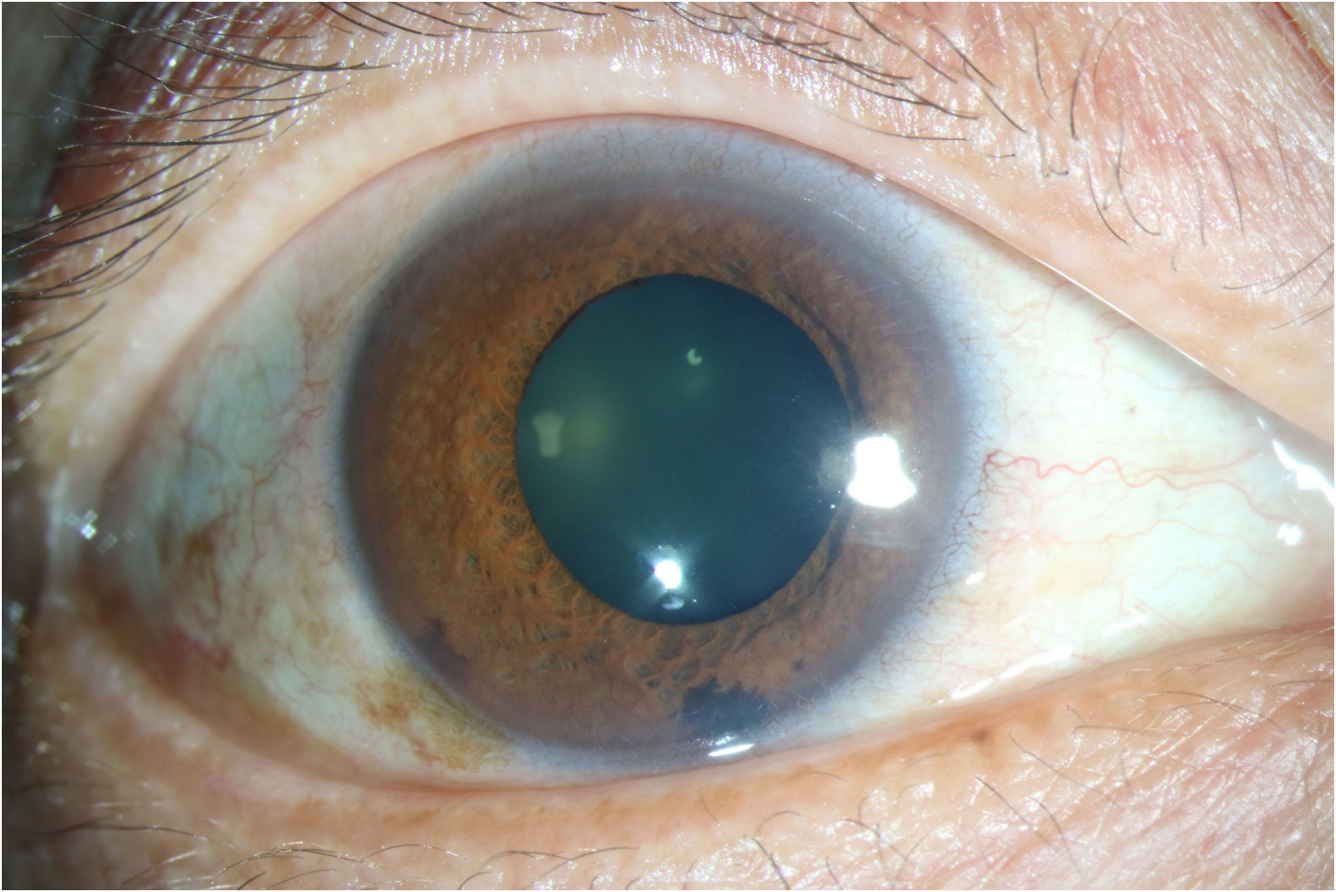
Anterior segment photography showing typical Iris depigmentation. Diffuse patchy iris depigmentation could be seen. The photography was captured from the right eye of a male patient aged 51.

**Figure 2:**
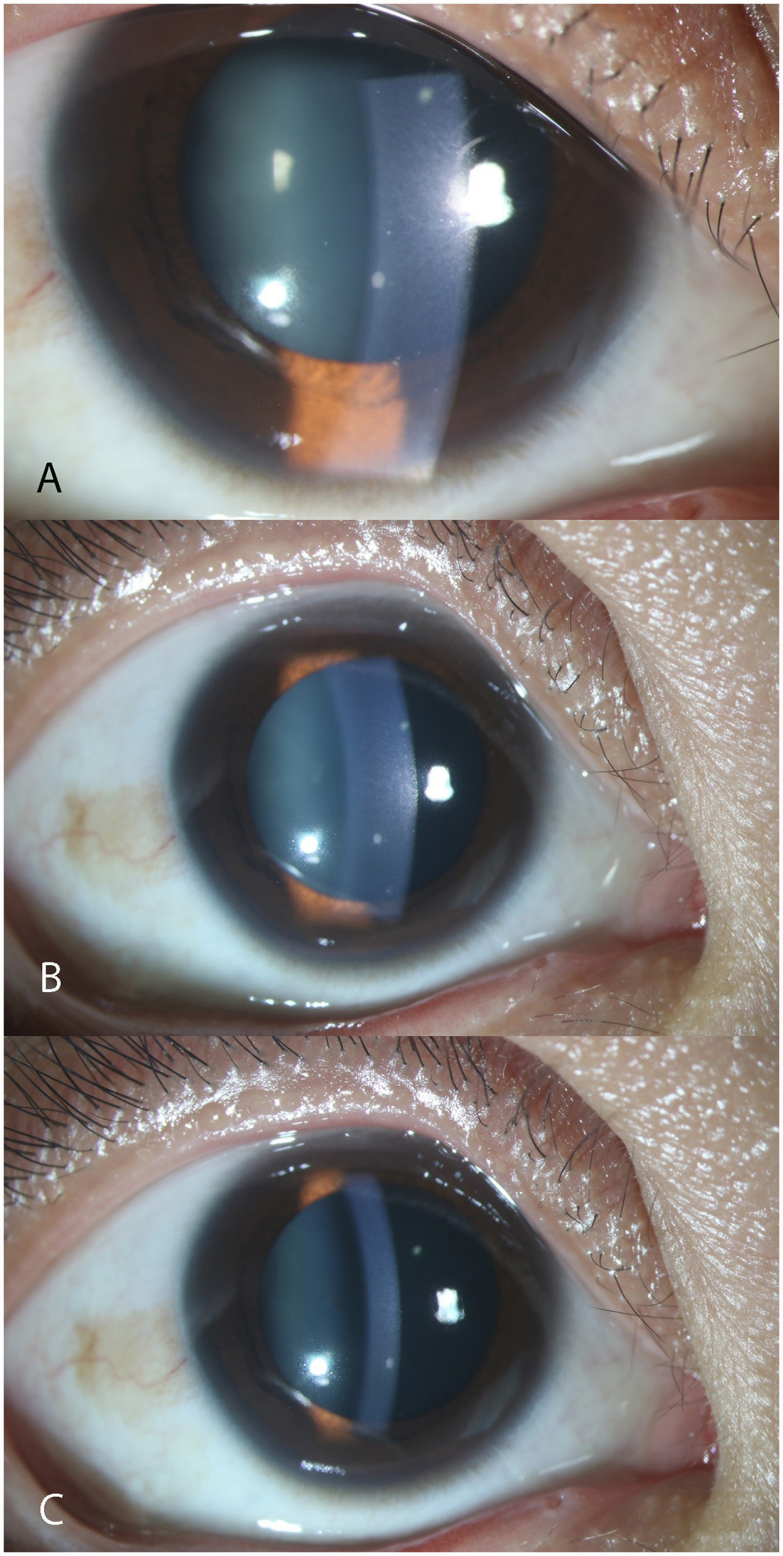
Anterior segment photography showing typical fresh keratic precipitates. The three photographs were captured from the right eye of the same female patient aged 26 during follow-up. From A to C, a clear process of KP reduction was reflected according to the timeline. However, the big mutton-fat KP still remained. **(A)** III-Degree. More than 10 fresh KPs in mutton-fat pattern can be seen on the lower middle endothelium under the slit-lamp. **(B)** II-Degree. **(C)** I-Degree. KP, keratic precipitate.

Totally 101 participants meeting the criteria above were included in the study. Apart from CMV, no other pathogen was screened positive in all samples. After paired aqueous humor/serum albumin concentration correction, 46 patients were tested CMV ratio higher than 0.40 and the mean ratio value was 1.78, and they were classified in CMV reactivation group. The other 55 patients whose CMV ratio between 0.00 and 0.40 (mean: 0.17) were classified in CMV latent group. Patients' characteristics were shown in Table 1. Typical iris depigmentation and the three degrees of fresh KPs were shown in Figures 1, 2.

**Table 1:** Demographics of 101 patients diagnosed Posner-Schlossman Syndrome.

	**CMV reactivation (*N* = 46)**	**CMV latent (*N* = 55)**	***P*-value**
Age (Years)	41.00 ± 13.54	38.23 ± 11.51	0.28
Gender (M/F)	25/21	34/21	0.45
Laterality (OD/OS)	23/23	24/31	0.52
Disease course (Months)	95.35 ± 77.67	49.99 ± 49.24	<0.001*
IOP (mmHg)	21.99 ± 9.14	24.36 ± 11.46	0.26
**Fresh KP**			
Total	44	47	0.09
III-degree^†^	4	10	0.17
II-degree	5	8	0.58
I-degree	35	29	<0.05*
Old KP	9/46, 19.57%	12/55, 21.82%	0.78
Iris depigmentation	36/46, 78.26%	32/55, 58.18%	<0.05*
Tyndall reaction	8/46	13/55	0.44
Anterior chamber cell	0	0	/
Mean CDR	0.57	0.46	<0.05*
Mean visual acuity^‡^	0.31	0.14	<0.05*
Mean loss rate of CEC	19.46%	10.86%	<0.001*
Mean CMV IgG correction ratio^§^	1.78	0.17	<0.05*
Mean previous corticosteroid dosage (Drops/day)	2.42	2.07	0.14
Mean previous IOP-lowering drugs dosage (Drops/day)	1.20	1.63	0.07
Mean Previous Antiviral Dosage (Drops/day)	0	0	/

*M, male; F, female; N, number; OD, oculus dexter; OS, oculus seminar*.

*CMV, cytomegalovirus; IOP, intraocular pressure; KP, keratic precipitate; CDR, cup-to-disc ratio; CEC, corneal endothelial cell*.

The bold values marked with * were statistically significant (P < 0.05).

† *0, no fresh KP in the anterior chamber; I, 1–5 KPs; II, 6–10 KPs; III, >10 KPs*.

‡ *Visual acuity was measured in logMAR (logMAR = logarithm of the minimum angle of resolution)*.

§ *Measured by paired cytomegalovirus IgG aqueous humor/serum correction ratio determined by a radial immunodiffusion technique, tested by Wang XL et al. (17)*.

The two groups shared similar age, gender ratio and laterality (*P* > 0.05). The initial IOP level and KP amount were similar in the two groups (*P* > 0.05). Patients in CMV reactivation group experienced a longer disease course than the latent group (*P* < 0.001), and had higher rate of iris depigmentation and larger CDR (*P* < 0.05). No cell was seen in the anterior chamber of both groups. The reactivation group lost 19.46% corneal endothelial cells in the affected eye compared to the other eye on a mean basis, while the latent only lost 10.86% (*P* < 0.001).

After a mean treatment period of 6.20 ± 4.36 weeks, patients' IOP and other anterior chamber inflammation manifestations were presented in Table 2.

**Table 2:** Ocular manifestations and medication at the end of follow-up (*N* = 101).

	**CMV reactivation (*N* = 46)**	**CMV latent (*N* = 55)**	***P*-value**
Treatment time (Weeks)	6.50 ± 4.67	5.95 ± 4.11	0.53
Cumulative ganciclovir drops (Drops)	181.70 ± 130.95	161.89 ± 110.66	0.41
**IOP (mmHg)**			
At the first visit	21.99 ± 9.14	24.36 ± 11.46	0.26
At the end of follow-up	19.83 ± 8.62	16.17 ± 4.95	<0.05*
*P*-value	0.25	<0.001*	
**Fresh KP**			
Total	15	16	0.70
III-degree^†^	0	4	0.06
II-degree	0	1	0.36
I-degree	15	11	0.15
Old KP	12/46, 26.09%	12/55, 21.82%	0.62
Tyndall reaction	0/46	2/55	0.19
Anterior chamber cell	0	0	/
Mean CDR	0.61	0.47	<0.001*
Mean current corticosteroids dosage (Drops/day)	1.13	0.87	0.30
Mean current IOP-lowering drug dosage (Drops/day)	0.87	0.95	0.73

*CMV, cytomegalovirus; IOP, intraocular pressure; KP, keratic precipitate; CDR, cup-to-disc ratio*.

The bold values marked with * were statistically significant (P < 0.05).

† *0, no fresh KP in the anterior chamber; I, 1-5 KPs; II, 6-10 KPs; III, >10 KPs*

The CDR rose faster in the reactivation group, from 0.57 to 0.61 (mean). The treatment period and cumulative 2% ganciclovir drops saw no significant difference in the two groups (*P* > 0.05). At the end of follow-up, IOP of the reactivation group remained at a similar level (*P* > 0.05), while in the CMV latent group IOP dropped significantly (*P* < 0.001). IOP of the reactivation group was significantly higher than the latent group (*P* < 0.05).

**Figure 3:**
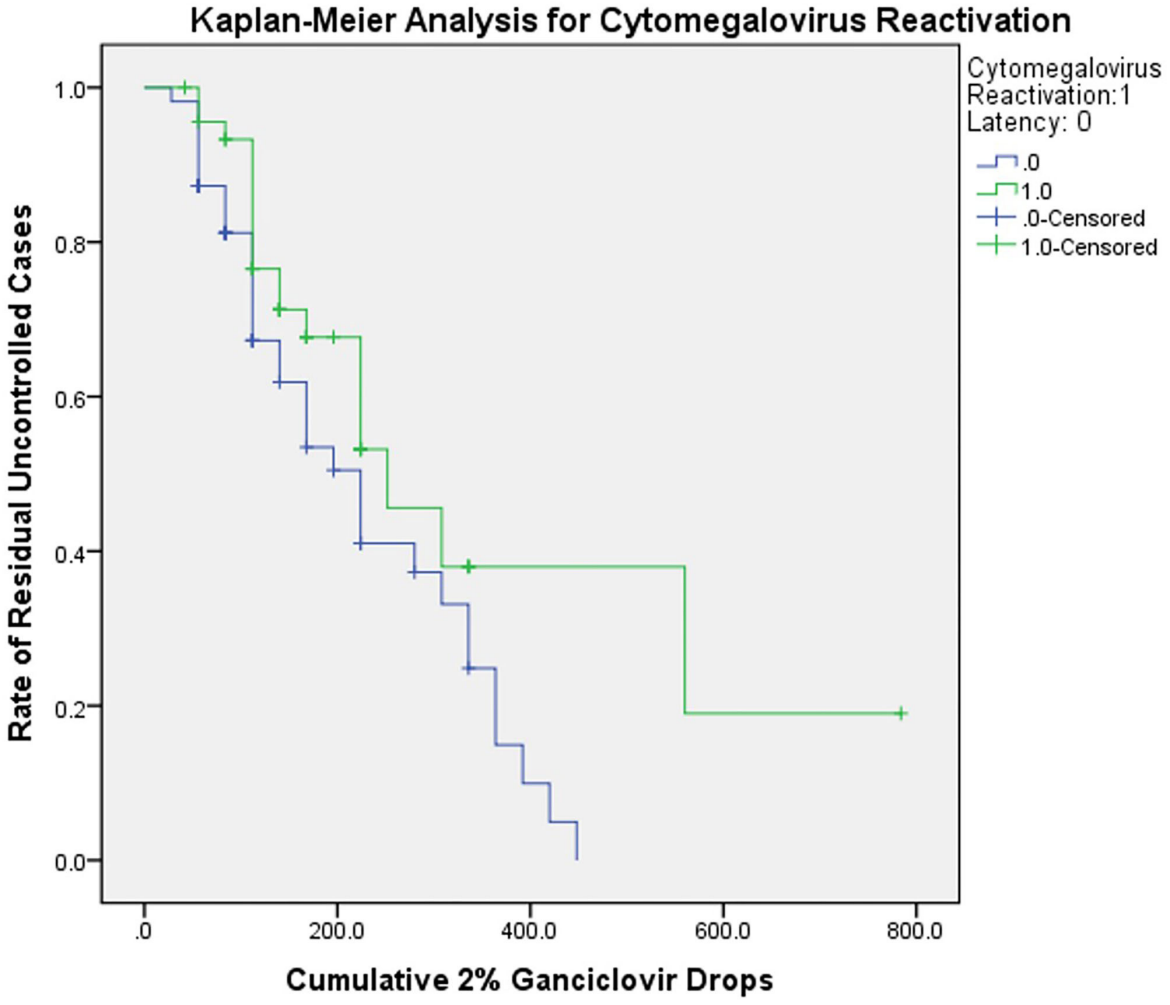
Kaplan-Meier analysis for CMV reactivation and latent group. The curve showed the relationship between cumulative ganciclovir dosage and rate of residual uncontrolled cases. The cumulative 2% ganciclovir drops were acquired by multiplying daily dosage and treatment period. The uncontrolled cases were cases whose IOP or anterior chamber inflammation still cannot be controlled even under the maximum ganciclovir dose predicted. The censored data indicated that actual cumulative doses were unknown in those cases (caused by loss of follow-up or withdrawal; all were predicted statistically). The curve reflected potential tendency in ganciclovir application. CMV, cytomegalovirus.

The results of survival analysis were shown in Figure 3. The predicted cumulative maximum 2% ganciclovir drops for complete control of relapses were 560.00 and 448.00 drops for the reactivation and latent group. The uncontrolled cases were cases whose IOP or anterior chamber inflammation still cannot be controlled even under the maximum ganciclovir dose predicted. The residual rate of uncontrolled cases was 0.19 ± 0.15 and 0, respectively (*P* < 0.05). After censoring and correction, for the CMV reactivation group, the median cumulative ganciclovir eye drops needed was 252.00 ± 50.71 drops (95% confidence interval: 152.60 to 351.40); for CMV latent group, the median was 224.00 ± 32.45 drops (95% confidence interval: 160.40 to 287.60), suggesting an estimated treatment course of 9 and 8 weeks.

## Discussion

Generally, 2% ganciclovir eye drops showed positive effect in controlling acute attack in PSS relapses both with and without CMV replication in our study. In addition, standardized and timely ganciclovir application helped patients reduce corticosteroid-dependence, which may benefit further prognosis and reduce the incidence of corticosteroid-induced glaucoma and cataract formation (19). Being convenient and effective, 2% ganciclovir eye drops deserve to be recommended to all PSS patients under high risk of recurrent CMV attack in clinical practice.

Markomichelakis NN et al. (20) extrapolated from the preceding data that ocular human CMV infection generally includes primary infection, reinfection with new strains, reactivation of latent infection, or persistent infection. The sudden increase of aqueous humor CMV IgG suggested recent CMV intraocular reactivation, which may result in the relapse. We speculated that relapses caused by CMV reactivation may happened frequently, for the reactivation group had significantly longer disease course. During acute attack, patients with CMV reactivation had poorer VA, which may result from corneal edema (21). The higher loss rate of CEC was also consistent with the endothelium damage pathogenesis (22, 23).

Elevated IOP, KPs and iris depigmentation were representative signs of acute PSS relapses (2). From the results above, we proposed whether innate relations existed between CMV ocular infection and either of these manifestations. KPs are corneal endothelial deposits associated with anterior chamber inflammation. KPs are commonly believed composed of aggregation of polymorphonuclear cells, lymphocytes, and epithelioid cells (24, 25). In our results, the rapid reduction of fresh KPs (mainly categorized in coin-shaped, mutton-fat, stellate and dendritic patterns) indicated the control of acute attack. However, the old KPs stayed unresponsive to either corticosteroids or ganciclovir, most of which contained pigment under the slit lamp. These long-existed pigmented precipitates were hard to eliminate completely either in the reactivation or in the latent group. Meanwhile, iris depigmentation was commonly seen in both groups. *In vivo* studies, CMV antigen was found first gathered at the root of iris, then distributed in the entire layers of iris, and last confined to iris in mice after intracameral injection from day 3 to day 10–12 (26). Markomichelakis also confirmed that iris stroma and pigmented epithelium were susceptible to CMV (20). It was speculated that iris pigmented epithelium might be under mild but continuous attack in CMV reactivation, since unlike acute iris depigmentation, no circulating pigment was seen in the anterior chamber (27). Our study supported that the IOP rise during acute attack of CMV infection may be partly resulted from the clogging of the trabecular meshwork with pigment deposition, as described by Tubal (27). Besides, it was also worth noting that the attack and control of anterior chamber inflammation should be based on the appearance and disappearance of fresh KPs, rather than pigmented ones.

With 2% ganciclovir eye drops, the mean daily dosage of corticosteroids and IOP-lowering drugs dropped even during the acute phase. Foreseeably, lower dosage would be needed to maintain a stable condition. In addition, the beginning of ganciclovir tapering should be considered cautiously. Since clinical manifestations of acute attacks varied individually, we recommended taking IOP peak, anterior chamber inflammation, fundus impairment, as well as the speed of uveitis control into consideration. Besides, patients' compliance and regular medication history are also important. Generally, an estimated dose of 252 drops and 224 drops is recommended to reactivation and latent patients respectively, which equals to a median course of 9 weeks and 8 weeks. A combination therapy of corticosteroids and ganciclovir is worth exploring in controlling acute CMV keratouveitis and PSS.

The reason why CMV replication was not detected in the latent group remained unknown. It was speculated that CMV may lurk in the anterior segment during relapses rather than circulating in aqueous humor, with iris and ciliary body mostly suspected (28). The primary cause of CMV latency was not fully discovered as well, since no topical or systematic immunosuppression was applied to these patients previously. However, aqueous humor antibody test was a convenient tool for assessing CMV infection. In our preliminary studies, aqueous humor CMV positive patients accounted for nearly 60% of the PSS population (18). Since CMV was an opportunistic infection pathogen in immunocompetent patients, aqueous humor antibody test may benefit those previously attacked or under high risk of relapse. These patients were often presented with long disease course, frequent relapses, old pigmented KPs on the endothelium, high percent of iris depigmentation, larger CDR and endothelial cell loss. It was predicted that the preventive 2% ganciclovir treatment in relapses without CMV reactivation would receive promising efficacy, for the residual rate was 0 as statistically estimated. A control group of latent patients without ganciclovir treatment needs to be supplemented.

Our study had several limitations. First, we did not include aqueous humor polymerase chain reaction results to confirm the replication of CMV. Though the aqueous humor/serum IgG correction ratio had a credible positive rate for CMV replication, it would be more exhaustive to combine the two tests together in evaluating acute attack. However, we were aiming at comparing the manifestations and the efficacy of 2% ganciclovir of relapses with/without CMV reactivation, the antibody correction ratio was comprehensive in reflecting recent viral replication. Second, the follow-up period was not long enough to observe midterm and long-term efficacy of ganciclovir. The end point of follow-up was determined by the control of acute anterior chamber inflammation and decrease of IOP, so was the cumulative ganciclovir dose calculation. The cumulative doses for acute relapses had already been estimated. We are extending our follow-ups and sample size as well to perform further explorations.

In conclusion, 2% ganciclovir eye drops showed promising effect in patients with CMV intraocular reactivation. The preventive and combined therapy of 2% ganciclovir are also recommended, which may shed a light on the control of PSS patients with historical CMV attack.

## Data Availability Statement

The raw data supporting the conclusions of this article will be made available by the authors, without undue reservation.

## Ethics Statement

The studies involving human participants were reviewed and approved by the Ethics Committee of Eye & ENT Hospital, Fudan University, Shanghai, China. The patients/participants provided their written informed consent to participate in this study. Written informed consent was obtained from the individual(s) for the publication of any potentially identifiable images or data included in this article.

## Author Contributions

QS carried out the study, analyzed data and wrote the main manuscript, and was a major contributor of the study. XK designed the study and reviewed the manuscript. XF and RZ participated in study-carried out and monitored the process. All authors have read and approved the final manuscript.

## Funding

This study was funded by the Western Medicine Guidance Project of Shanghai Committee of Science and Technology (19411961600), the Experimental Animal Research Project of Shanghai Science and Technology (201409006600), and the Double Excellent Project of EENT Hospital (SYB202003). The authors were funded by the Surface Project of National Natural Science Foundation of China (81770922 and 82070957). The funders had no role in study design, data collection, analysis and interpretation, decision to publish, or preparation of the manuscript.

## Conflict of Interest

The authors declare that the research was conducted in the absence of any commercial or financial relationships that could be construed as a potential conflict of interest.

## Publisher's Note

All claims expressed in this article are solely those of the authors and do not necessarily represent those of their affiliated organizations, or those of the publisher, the editors and the reviewers. Any product that may be evaluated in this article, or claim that may be made by its manufacturer, is not guaranteed or endorsed by the publisher.
